# Electrochemical Crosslinking of Alginate—Towards Doped Carbons for Oxygen Reduction

**DOI:** 10.3390/polym15153169

**Published:** 2023-07-26

**Authors:** Jelena Rupar, Armin Hrnjić, Snežana Uskoković-Marković, Danica Bajuk-Bogdanović, Maja Milojević-Rakić, Nemanja Gavrilov, Aleksandra Janošević Ležaić

**Affiliations:** 1 Faculty of Pharmacy, University of Belgrade, 11221 Belgrade, Serbia; jelena.rupar@pharmacy.bg.ac.rs (J.R.); snezana.uskokovic@pharmacy.bg.ac.rs (S.U.-M.); aleksandra.janosevic@pharmacy.bg.ac.rs (A.J.L.); 2 Laboratory for Electrocatalysis, Department for Materials Chemistry, National Institute of Chemistry, Ljubljana, SI-1001 Ljubljana, Slovenia; armin.hrnjic@ki.si; 3 Faculty of Physical Chemistry, University of Belgrade, 11158 Belgrade, Serbia; danabb@ffh.bg.ac.rs (D.B.-B.); maja@ffh.bg.ac.rs (M.M.-R.)

**Keywords:** oxygen reduction, catalysis, nanoparticles, alginate, doping

## Abstract

Electrochemical crosslinking of alginate strands by in situ iron oxidation was explored using a potentiostatic regime. Carbon-based materials co-doped with iron, nitrogen, and/or sulfur were prepared via electrolyte composition variation with a nitrogen-rich compound (rivanol) or through post-treatments with sodium sulfide. Nanometer-sized iron particles were confirmed by transmission and field emission scanning electron microscopy in all samples as a consequence of the homogeneous dispersion of iron in the alginate scaffold and its concomitant growth-limiting effect of alginate chains. Raman spectra confirmed a rise in structural disorder with rivanol/Na_2_S treatment, which points to more defect sites and edges known to be active sites for oxygen reduction. Fourier transform infrared (FTIR) spectra confirmed the presence of different iron, nitrogen, and sulfur species, with a marked difference between Na_2_S treated/untreated samples. The most positive onset potential (−0.26 V vs. saturated calomel electrode, SCE) was evidenced for the sample co-doped with N, S, and Fe, surpassing the activity of those with single and/or double doping. The mechanism of oxygen reduction in 0.1 M KOH was dominated by the 2e^−^ reduction pathway at low overpotentials and shifted towards complete 4e^−^ reduction at the most negative explored values. The presented results put forward electrochemically formed alginate gels functionalized by homogeneously dispersed multivalent cations as an excellent starting point in nanomaterial design and engineering.

## 1. Introduction

Electrocatalysis is in the spotlight of an emerging search for electroactive materials aiming to solve energy-related issues. Oxygen reduction reaction (ORR), as the limiting fuel cell reaction, requires constant material advances aimed at finding a proper replacement for carbon-supported platinum catalysts [1]. Part of the search is orientated towards sustainable resources or exhausted materials [2,3] and targets biobased carbonaceous materials [4], either waste-originated or readily available in the environment [5].

Carbon-based catalysts with a transition metal component possess desirable properties with regards to ORR at most pH values alongside suitable corrosion resistance and decent conductivity [6]. Among prospective non-noble metals, iron-based catalysts take the center stage due to iron abundance, low environmental impact, and the ability to provide high conductivity in carbon-based catalysts [7]. Thus, iron-based catalysts present a main focal point among non-precious metal-based materials for the ORR [8,9,10,11]. Good catalytic activity has been shown when the iron is stabilized by a scaffold made up of carbon, nitrogen, and/or sulfur atoms. The main idea behind these efforts is to mimic Fe centers in enzymes, which often have similar active center structures. The known complex structures of porphyrins/phthalocyanines are recognized as promising metal–organic catalysts [12], but the high price and tedious synthesis inhibited their further development for practical applications [13]. The reasonable activity of iron-centered structures can be explained by induced charge redistribution on the surrounding heteroatoms, which can then geometrically fit oxygen adsorption and concurrent charge relocation so as to weaken the double bond and enable for reduction/protonation [14,15]. These Fe-centered structures have a certain stability due to strong Fe interaction with neighboring atoms, and they also possess structure-directing qualities as they prevent the agglomeration of particles. Heteroatoms (C, N, S, P) form a strong bond with iron and show good charge transfer properties which can, in connection with their good chemical stability and suitable conductivity, put them forward as good catalytic supports for other electrocatalysts [16]. Doped carbons usually rely on N doping, and recently investigated iron-containing nanostructured samples derived by pyrolysis of chitosan/melamine nanostructures are selected as promising high-performance ORR catalysts [13]. Electroactive iron-doped carbon preparations may be obtained via interesting routes such as the sintering of polyethersulfone membranes, with oxygen groups put onto the surface with subsequent nitric acid treatment [17]. A readily adopted route to prepare these heterostructures is to use macrocyclic molecules of metal–organic frameworks (MOFs) with already existing Fe–heteroatom bonds [18,19,20].

Recent advances in the field are noteworthy, as summarized in recent reviews [21]. This goal is mainly achieved by intimate doping of Fe atoms inside a carbon matrix which alters the local electronic structure and can then serve as an oxygen reduction center. Tailoring the synthesis procedure towards specific carbon–Fe structural units that possess high catalytic activity and selectivity towards direct 4e^−^ reduction is still challenging. Some research suggests that, under alkaline conditions, properly coordinated Fe^2+^ sites can effectively reduce the HO_2_^−^ intermediate towards OH^−^ and complete the second 2e^−^ reduction in the oxygen reduction pathway [22]. Moreover, the excess of OH^−^ ions can saturate iron surfaces, preventing O_2_ from reaching the active center where it is reduced [23]. However, precise control in the preparation of uniform and uniquely active Fe–carbon centers that are stable under alkaline conditions is still a challenge which, if/when accomplished, can provide a definite answer to the individual catalytic steps and guide further catalyst improvement.

Therefore, the ease of synthesis and biobased precursors are important aspects, since efficient materials may sometimes be gained through numerous synthesis steps employing costly carbon sources. The composition/structure of bio-precursors can be tailored with supreme precision if electrochemically controlled synthesis is applied [24]. This is predominantly related to the introduction of heterogeneous atoms that are needed for charge delocalization and improvements in catalytic activity [25,26]. The doping procedure can be elegantly accomplished through the introduction of different additives into the electrolytic cell during electrode-deposited precursor formation [27]. The developed surface is another parameter necessary for adequate catalytic performance, which can either be addressed in pre-carbonization synthesis (hydroxide activation [28]) or within a carbonization procedure where local thermal events can produce sufficiently high specific surface area (around 1000 m^2^ g^−1^) and pore structure such that they are suitable for electroactive applications [27]. Additionally, well-organized bio-oligomers/polymers are easily cross-linked around metal cations that can additionally act as surface developers while, at the same time, eliminating the need for templating techniques. Among bio-derived precursors, alginate is found to be a particularly interesting polyanionic polymer that originates from readily available algae/bacteria [29]. Interconnected units of β-D-mannuronate and α-L-guluronate are bridged via glycosidic bonds. Na/K alginate salts are viscous liquids undergoing sol–gel transitions in the presence of di-/trivalent cations and have versatile applications [29]. For instance, Guo et al. prepared hierarchical alginate-derived carbons for Li storage with remarkable electrochemical performance, upstaging graphite anodes [30]. So far, there have been several reports dealing with alginate precursors prepared by chemical methods [31,32], spinning procedures [33,34,35], and freeze-drying [36,37] for subsequent catalytic ORR applications. Thermal treatment was conducted in a wide temperature range (400−1000 °C) using metal dopants (Co [31,35,36,38], Cu [32], Ni [36], and Fe [37]) and nitrogen as a heteroatom. 

Motivated by recent work employing zinc alginate-based carbons in supercapacitors [27], we here propose a synthesis modification for hydrogel precursors to produce functional nanocarbons for catalytic applications. To evaluate the role of doping in overall catalytic performance, we propose a novel, straightforward, electrochemical synthesis procedure that addresses all important parameters for nanocarbon production for ORR. The proposed Fe electrode-assisted gelling procedure enables the sol–gel formation of alginate hydrogel. The gel is stabilized by electrostatic interactions of Fe–uronic alginate constituents [29]. These intermolecular interactions are susceptible to ion exchange/release, enabling efficient co-doping with additional heteroatoms. To differentiate the benefits of each heteroatom to material catalytic performance for ORR, a detailed characterization of iron-, nitrogen-, and sulfur-doped carbons is comprehensively addressed.

## 2. Materials and Methods

### 2.1. Nanocomposite Preparation

Sodium alginate (Na-alg) (M = 300,000–350,000 g mol^−1^) was supplied by Carl Roth (Karlsruhe, Germany). All other chemicals were of analytical grade, as was ultrapure MiliQ water.

Electrochemical synthesis of iron alginate (Fe-alg) samples was performed according to the previously designed procedure [27]. Briefly, the thin Fe electrode strip was placed as an anode ion source while the Cu electrode was used as the cathode. Gel formation proceeds in the immediate vicinity of the Fe electrode surface as formed ferric ions diffusing away from the electrode, enable crosslinking of nearby alginate units with a concomitant rise in viscosity. The resulting gel possesses a pale orange color and adheres to the Fe electrode. An HQ Power PS3003 Lab Power Supply was used to control the oxidation potential of 1.5 V in a 30 min run period. An electrolyte containing 2% Na-alg was used for Fe-alg gel sample formation. After washing out the excess alginate, the gel was removed from the electrode. To introduce nitrogen, rivanol (ethacridine lactate, extra pure, 99.9% Centrohem, Stara Pazova, Serbia) precursor was added to an alginate-containing electrolyte, which resulted in a gel Fe-alg-N sample. A sulfur-containing set of samples, Fe-S-alg and Fe-S-alg-N, was obtained through 0.5 M Na_2_S (p.a. Sigma-Aldrich, St. Louis, MI, USA) aqueous treatment of freshly prepared Fe-alg and Fe-alg-N gels for 2 h. Prepared gels were first dried at 60 °C and subsequently carbonized in argon for 2 h at 700 °C with a 10 °C/min heating rate in a tubular furnace. The resulting functionalized carbons are denoted as C/Fe and C/Fe.N (from Fe-alg and Fe-alg-N gels), while C/Fe.S and C/Fe.S.N originated from Fe-S-alg and Fe-S-alg-N precursors in that order.

### 2.2. Characterization

A DXR Raman micro-spectrometer (Thermo Scientific, Waltham, MA, USA) was used for Raman spectra collection. The 532 nm laser was employed with a power output of 2.0 mW, 10 × 10 s scan periods using 900 lines/mm grating, and a 50 μm aperture. 

For additional spectral analysis, Fourier transform infrared spectra were recorded using a KBr pellet technique in the 4000–400 cm^−1^ range. The 32 scans per spectrum were collected, enabling a 4 cm^−1^ resolution on the Nicolet iS20 spectrometer (Thermo Scientific, Waltham, MA, USA). 

The morphology of prepared samples was analyzed using a field emission scanning electron microscope (FESEM) (JSM-6700F (JEOL)) and a Phenom ProX equipped with an energy-dispersive X-ray spectrometer (EDX) (Thermo Scientific, Waltham, MA, USA). 

To confirm the nanostructures of synthesized samples, the transmission mode was also employed using an ultra-high resolution imaging H-8100 microscope (Hitachi Ltd., Chiyoda, Tokyo, Japan) with a LaB_6_ thermionic emission gun.

### 2.3. Electrochemical Measurements

A glassy carbon disc (GC) with a 5 mm diameter was covered by a thin homogeneous film and used for electrochemical testing as follows. To ensure homogeneity prior to catalytic ink preparation, samples were pulverized in an agate mortar. An ethanol/water/Nafion (Sigma-Aldrich, Louis, MI, USA) mixture was added to 5 mg of the sample and sonicated for 30 min to prepare the ink suspension used for thin film preparation. Twenty microliters of the ink was drop-casted on the GC disc (0.19625 cm^−2^) and the solvent was evaporated to reach a specific mass of material equal to 500 μg cm^−2^. Cyclic voltammetry (CV) was used to assess the activity of materials towards oxygen reduction reaction (ORR) in a standard three-electrode cell with Pt wire and a saturated calomel electrode (SCE) serving as the counter and reference electrodes, respectively. An alkaline 0.1 M KOH aqueous solution was used throughout ORR testing, with an Ivium VO1107 potentiostat/galvanostat driving the overpotential and measuring the current response. Oxygen concentration was kept constant by purging the solution for 15 min before the measurements and was maintained by a steady flow during the measurement itself. The baseline was recorded after the ORR measurement in a nitrogen-purged solution. All measurements were performed at ambient pressure and temperature with a rotating disk electrode (RDE) at 600, 900, 1200, 1800, and 2400 rpm using a PINE rotator. The apparent number of exchanged electrons per oxygen molecule was determined by employing Koutecký–Levich analysis (K–L) as shown in Equation (1):

(1) 1im=1ik+1Bω12,

where *i_m_*, *i_k_*, *B*, and *ω* represent the measured current, the kinetic current associated with the electrochemical reaction, the Levich constant, and the angular rotation rate, respectively.

## 3. Results

### 3.1. Microscopy

Chemically crosslinked Fe^3+^-alginate beads have been shown recently as a model system for <10 nm nanoparticle generation [39]. The electrochemically generated Fe^3+^ cations can also promote the sol–gel transition of alginate, resulting in a thin Fe-alg layer. After carbonization of the Fe-alg sample, the expected monodispersion of iron nanoparticles in a carbon matrix was confirmed by TEM within a similar size range.

**Figure 1:**
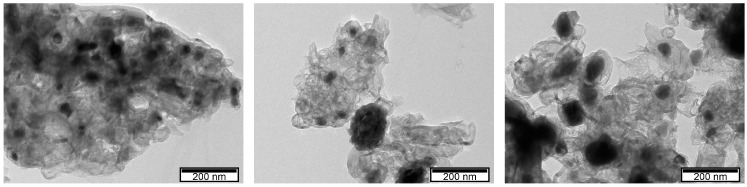
TEM images of C/Fe after carbonization and before the doping process.

Figure 1 reveals dozens of nanoparticles, between 20 and 40 nm in size, dispersed in a carbon matrix that shows a layered fibrous structure. Some coalescence and aggregation between particles is seen and may be the result of the stacking of alginate layers.

**Figure 2:**
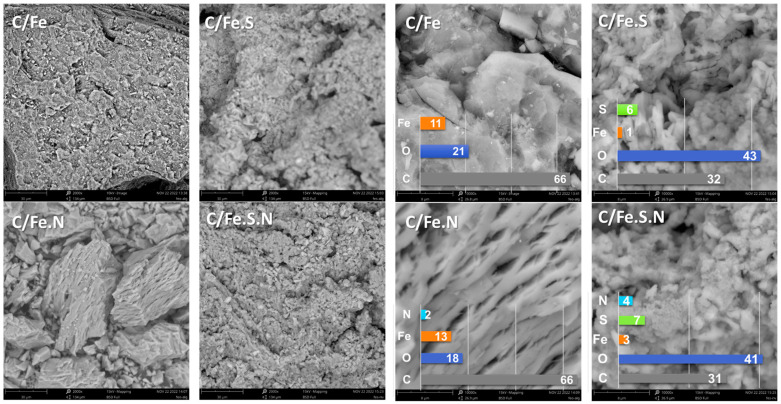
SEM micrographs with 2000× (**left**) and 10,000× magnification (**right**) and associated EDS results (atomic %) for tested carbons.

The SEM images with EDS analysis are given in Figure 2. A well-resolved planar structure is expected for alginate-derived carbons [40] and can be clearly seen for C/Fe and C/Fe.N samples, where a neatly connected alginate network was preserved after carbonization (Figure 2). The layered structure may be seen at different imaging angles and is best identified for C/Fe.N (Figure 2, left). The bright spots seen on the right of Figure 2 attest to some clustering of nanoparticles on the surface and edges where alginate coordination is not sufficient to stop particle agglomeration. Sodium sulfide treatment led to a loss of long-range ordering in the laminar structure in S- and S/N-containing carbons, as seen in Figure 2. The most likely effect is sodium sulfide-induced [29] partial Fe leaching from the gels, resulting in gel swelling and local chain disentanglement upon iron removal from the intertwined structure and the subsequent partial granular morphology in C/Fe.S and C/Fe.S.N samples. Iron leaching from the gel structure is reaffirmed when looking at the EDS measurements, where a decrease in iron content is seen for C/Fe.S.N. Loss of iron is pronounced for C/Fe.S and C/Fe.S.N, which might indicate that the presence of Na_2_S inhibits gel rigidity and helps subsequent leaching. A directing role can be seen through the porous, flaky morphology obtained by nitrogen doping in C/Fe.S.N, which is somewhat different from the tubular/thread formations seen in C/Fe.S. 

Significantly lower iron and carbon content was detected after the sulfide treatment of precursor gels in C/Fe.S and C/Fe.S.N relative to C/Fe and C/Fe.N samples (Figure 2, right). Treatment with Na_2_S not only reduced iron ions but concomitantly disentangled alginate chains. This led to the loss of gel rigidity and, more importantly, exposed more of the oxygen groups responsible for Fe^2+^ coordination. As for the rise in oxygen content, Na_2_S treatment induced/catalyzed carbon burn-off, and the rise in relative oxygen content was due to the decrease in carbon, iron, and nitrogen content. EDS results (included on the right of Figure 2) confirm that these samples are highly susceptible to experimental fine-tuning as there is a definite distinction between the structure/composition of prepared doped carbons.

**Figure 3:**
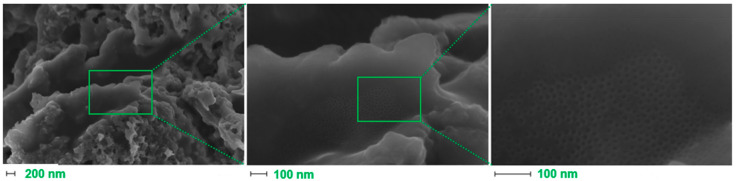
FE-SEM images of the C/Fe.S.N sample under different magnifications.

**Figure 4:**
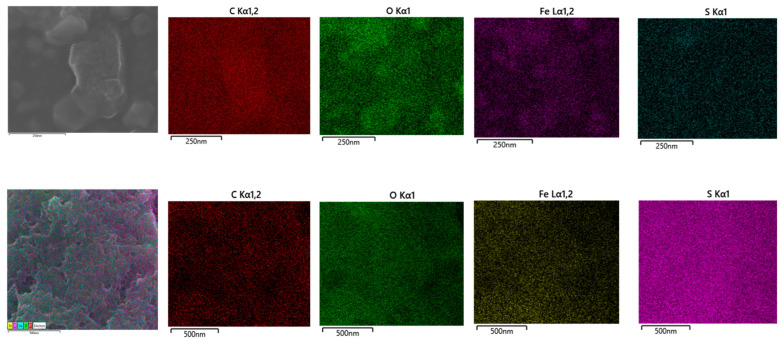
Selected elemental distribution for C/Fe.S (**top**) and C/Fe.S.N (**bottom**).

FE-SEM (Figure 3) was further used to assess the fine structure of C/Fe.S.N as a sample with the intended heteroatomic composition. High porosity and a large number of edges are clearly seen with increasing magnification. The homogeneous dispersion of hundreds of <10 nm nanoparticles is also witnessed in the last image. These images validate the proposed method as a viable means of preparing uniform nanoparticles of different metal–oxygen/sulfur species (Figure 4).

### 3.2. Raman and FTIR Spectroscopy

**Figure 5:**
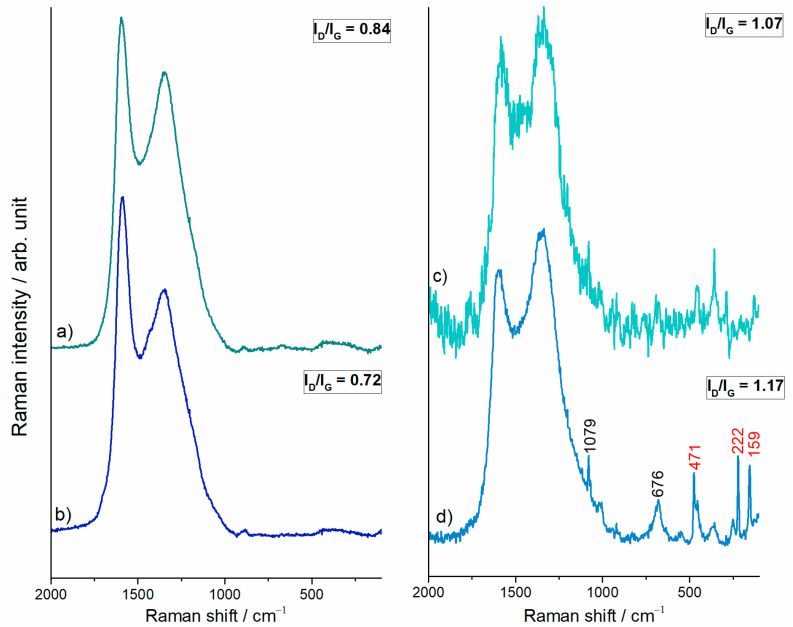
Averaged Raman spectra of (a) C/Fe (4 records), (b) C/Fe.N (4 records), (c) C/Fe.S (16 records), and (d) C/Fe.S.N (16 records).

**Figure 6:**
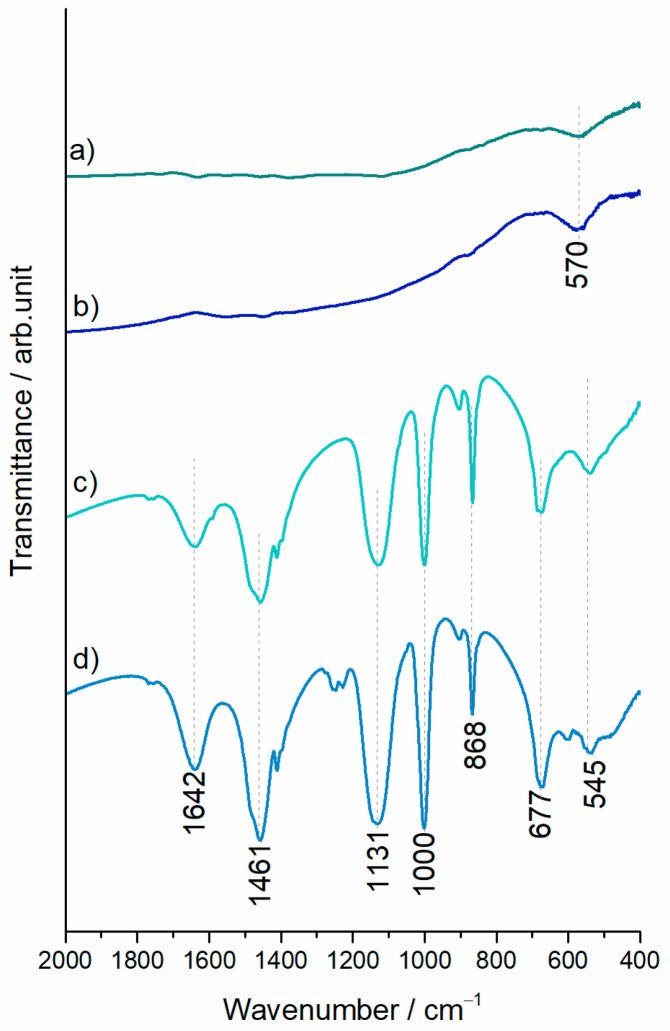
FTIR spectra of (a) C/Fe (b) C/Fe.N, (c) C/Fe.S, and (d) C/Fe.S.N.

As can be seen from the Raman spectra of investigated samples in Figure 5, thermal treatment of precursor gels at 700 °C produced carbon materials. Namely, the bands characteristic of carbonaceous materials, the G and D bands, can be seen at ∼1590 cm^−1^ and ∼1345 cm^−1^, respectively, due to sp^2^ hybridization. The intensity ratio of the two characteristic bands (I_D_/I_G_) increased in a sulfur-containing set of samples, as shown in Figure 5. Katagiri et al. suggested that the D band originates from carbon network disruption or edging [41]. The intensification of the Raman D band in C/Fe.S and C/Fe.S.N spectra (Figure 5c,d) is in line with local chain disentanglement in SEM micrographs (Figure 1) and confirms the carbon framework discontinuities. The synthesis/doping procedure introduced heteroatoms, which were detected as additional, non-carbon components in Raman spectra below 1000 cm^−1^ for the C/Fe and C/Fe.N samples. The FTIR spectra given in Figure 6 suggest the presence of iron (II, III) oxides (magnetite), which is demonstrated by a broad band at around 570 cm^−1^. The sulfide-treated C/Fe.S and C/Fe.S.N carbons are characterized by Raman spectra with bands in the region up to 1000 cm^−1^ where a substantial number of narrow vibrations were recorded, mainly for the C/Fe.S.N sample. Interestingly, the most prominent vibrations are positioned at 471, 222, and 159 cm^−1^, which is characteristic of elemental sulfur. Carbonate group vibrations are also resolved at around 1079 cm^−1^, as well as the magnetite band at 676 cm^−1^. The FTIR spectra of both C/Fe.S and C/Fe.S.N samples are comparable with carbonate (1461 and 868 cm^−1^) and thiosulfate (1131, 1000, and 667 cm^−1^) group vibrations [42].

### 3.3. Electrochemistry

**Figure 7:**
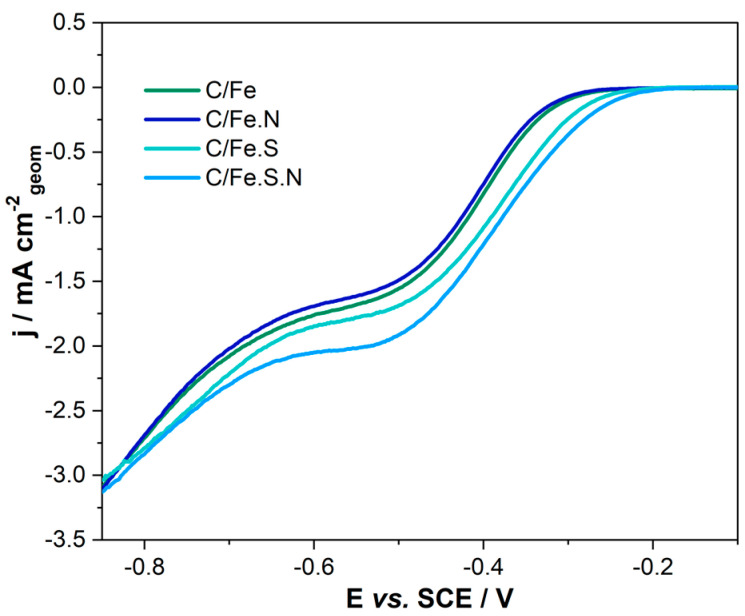
Background-corrected anodic scans of CV for tested doped carbons.

Alkaline media presents a less aggressive medium to catalysts with ORR kinetics, with less overpotential required in alkaline media than acidic media. However, highly basic conditions appear detrimental for ORR on Fe–carbon catalysts. Namely, recent research suggests that the excess OH^−^ ions adsorbed on iron prevent it from participating in the reduction and essentially serve as a catalytic poison [23]. To assess catalytic activity towards the oxygen reduction reaction, we employed cyclic voltammetry in a 0.1 M KOH solution saturated with oxygen. Observed differences in onset potential and current densities are seen in Figure 7, where only anodic scans of CV are shown (1200 rpm, 20 mV s^−1^).

The most positive onset potential (−0.26 V vs. SCE), as well as the highest current density in the whole potential window, was witnessed for C/Fe.S.N. Its activity is closely matched by C/Fe.S, which exhibited a 20 mV negatively shifted onset (−0.28 V vs. SCE) and lower current density. The results obtained here closely match those reported by Fang et al. [43] for FeS/FeS_2_/rGO composites, as well as those reported by Ai et al. [44] for N/S co-doped graphene, which reported higher current densities on the order of several mA cm^−2^. Comparison points to the positive role of nitrogen doping of the supporting carbon [45] on activity towards ORR, especially considering the similar chemical composition (Fe, S, C, and O content). Further comparison versus non-treated samples (C/Fe and C/Fe.N) reveals that the leaching of iron ions from the intertwined alginate precursor structure allows for further reaction with excess sulfide ions. This leads to the formation of different iron/sulfur species during the carbonization process, as seen by Raman and FTIR analyses. Additionally, a homogeneous dispersion of nanoparticles, as witnessed in the FE-SEM image of C/Fe.S.N, is possibly the reason for this high activity. The doping mechanism thus leads to improved activity (60–70 mV positively shifted onsets), better dispersion, as reported earlier [46,47], and smaller particles that can optimize ORR processes, which is in line with previous studies [48]. 

Iron oxide, dispersed in N-doped/undoped alginate-directed carbon networks, displays somewhat lower onset potential and current density compared to C/Fe.S and C/Fe.S.N but is on par with other similar materials [49]. The negligible difference in onset potential (−0.32 V vs. −0.33 V) and current density for C/Fe and C/Fe.N points to similarities between these materials. Higher Fe content in both materials (>30 wt.% for C/Fe and C/Fe.N, Table 1) might result in higher particle coalescence, blocking some of the active sites and leading to a concurrent loss of conductivity. The addition of rivanol as a nitrogen source seems to have little influence on the activity of iron oxide(s) towards ORR, a markedly different effect than that seen for sulfide-treated samples where addition had a promoting effect. The difference in nitrogen doping level might be the reason (<2 at.% for C/Fe and ~4 at.% for C/Fe.S.N) for this discrepancy (Table 1). To discern the underlying mechanism of the ORR, we conducted Koutecký–Levich analysis in a wide potential window.

**Figure 8:**
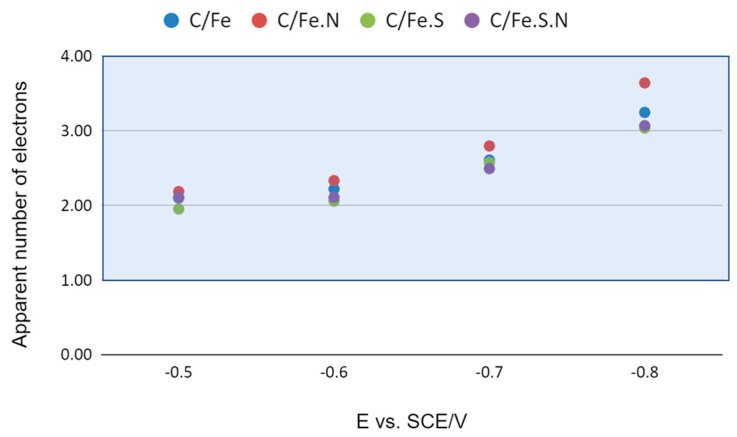
Apparent number of exchanged electrons in the ORR process for tested samples according to K–L analysis.

The results are graphically presented in Figure 8 and reveal that oxygen reduction proceeds through initial two-electron reduction in all samples. At more negative potentials, the mechanism changes as the number of apparently exchanged electrons rises to over three. This can be a sign of change towards direct four-electron reduction or, alternatively, to disproportionation of 2e^−^ products entering another 2e^−^ reduction cycle, giving rise to a higher apparent number of exchanged electrons. An identical mechanism shift from two- to four-electron reduction is common in many similar systems [50,51,52]. We consider the 3e^−^ reduction to be the result of the superposition, essentially the parallel occurrence of the mechanism with two and four electrons at more negative potentials.

*In situ* electrochemical intertwining of iron ions in an alginate precursor led to homogeneously dispersed nanoparticles after carbonization. The co-doping of polymer precursors with nitrogen and sulfur led to the most promising catalyst for ORR after carbonization, as seen by the 4e^−^ mechanism almost being reached at more negative potentials. 

## 4. Conclusions

Straightforward electrochemical preparation of homogeneously dispersed iron nanoparticles inside an alginate matrix is herein described through successful in situ doping with nitrogen. Post-treatment with sodium sulfide proved effective in co-doping the formed gel with sulfur. Uniform arrays of nanoparticles were formed when ethacridine lactate was introduced in the initial alginate solution, as witnessed by FE-SEM images. Post-treatment also resulted in lower iron content and the change from a laminar to globular morphology via the untangling of parts of the alginate net. Carbonized samples were tested as catalysts for oxygen reduction in an alkaline solution, which showed that activity rose in the following sequence: C/Fe.S.N > C/Fe.S > C/Fe.N ≈ C/Fe. Co-doping with nitrogen and sulfur, in addition to iron, proved advantageous over single heteroatom doping. The onset potential for C/Fe.S.N amounted to −0.26 vs. SCE, close to values reported for materials with similar composition. Selectivity was the same in all tested materials and reached the desired complete 4e^−^ reduction at high negative potentials. The results presented here offer some insights into a green and inexpensive way of designing an effective nano-structured non-noble catalyst for oxygen reduction. 

## Figures and Tables

**Table 1:** EDS results of surface elemental composition in at.%.

Element	C/Fe		C/Fe.N		C/Fe.S		C/Fe.S.N	
C	65.7	44.0	65.8	43.0	31.9	22.0	30.6	20.0
O	21.3	19.0	17.6	15.0	42.6	39.0	40.9	36.0
Fe	11.1	35.0	12.8	38.0	1.4	4.0	2.7	8.0
S	0.4		0.3		6.0	11.0	7.5	13.0
N			2.3	2.0			3.9	3.0

## Data Availability

Data are contained within the article.
